# Supplementary material for: A novel adenovirus 19K/IX protein enhances infection by attenuating proteasomal degradation of tyrosine-ubiquitinated capsid protein IX
Source: mBio. 2026 Oct 14;17(11):e01683-26. doi: 10.1128/mbio.01683-26 (PMC13753832; doi:10.1128/mbio.01683-26)
Supplement: Supplementary file 1 [file mbio.01683-26-s0001.pdf]

Supplementary Figure Legends

Fig S1. **Expression and subcellular localization of the pIX and 19K/IX proteins. (A)** Lack of 19K/IX reduces pIX expression. A549 cells were infected (MOI = 5) with Wt or Stop^M, and whole-cell lysate (WCL) was prepared at 12, 16, 20, and 24 hpi. WCLs were analyzed by western blotting (WB) with the anti-HA, anti-Flag, anti-E1A, anti-fiber, and anti-tubulin antibodies. **(B)** pIX induces nuclear inclusions. Immunofluorescence (IF) of A549 cells infected (MOI = 5) with Wt or Stop^M at 24 hpi. pIX-HA was stained with the HA antibody, and nuclei were stained with DAPI. Scale bar = 10 μm. **(C)** pIX is relocalizing 19K/IX into nuclear inclusions. IF in A549 cells transiently transfected with plasmids expressing HA-pIX, V5-19K/IX, or both proteins at 24 hpt. The proteins were stained with the HA and V5 antibodies, and nuclei were stained with DAPI. Scale bar = 10 μm. **(D)** The same IF as in panel C, but performed in H1299 cells at 24 hpt. **(E)** Epitope tagging does not change 19K/IX localization. IF in A549 cells transiently transfected with plasmids expressing HA-pIX, V5-19K/IX, or both proteins at 24 hpt. The proteins were stained with the HA and V5 antibodies, and nuclei were stained with DAPI. Scale bar = 10 μm.

Fig S2. pIX is ubiquitinated in infected cells. H1299 cells transiently expressing His-ubiquitin (His-Ubi) were infected with Wt or Stop^M (MOI = 50) for 24 h. Ni-NTA affinity-purified His-Ubi-conjugated proteins were separated by WB and analyzed with the indicated antibodies. Ubiquitinated pIX species are indicated; a red asterisk indicates Flag-19K/IX-HA.

Fig S3. pIX is not ubiquitinated at serine and threonine residues. (A) Amino acid alignment (Clustal Omega Multiple Sequence Alignment) of pIX proteins belonging to different HAdV types (HAdV-C5 (P03281), HAdV-A12 (P03284), HAdV-F41 (P32539), HAdV-G52 (A0MK45), HAdV-D37 (B2VQE5), HAdV-B7 (P68971), HAdV-E4 (Q2KSG2)). Conserved tyrosine (Y) residues (Y14 and Y49 in HAdV-C5) are boxed in red. Non-conserved lysine (K) residues (K99 and K132 in HAdV-C5) are boxed in green. **(B)** Schematic illustration of the serine (S) and threonine (T) residues in HAdV-C5 pIX. Mutations of the respective S and T residues to alanine (S/T>A) in the individual pIX proteins are shown. **(C)** Serine and threonine mutations do not change pIX ubiquitination. H1299 cells transfected with HA-Ubi and individual V5-pIX (panel B) encoding plasmids. The V5-tagged proteins were immunopurified and probed with anti-HA (green, detects ubiquitinated proteins) and anti-V5 (red, detects pIX). **(D)** V5 epitope tag (NH2-G**K**PIPNPLLGLDST-COOH) is not ubiquitinated despite the presence of the lysine (marked in bold) residue. V5-pIX(Wt) and irrelevant V5-epitope tag containing HAdV-C5 protein pVII(K26R/K27R) (referred to as V5-pVII) were immunopurified (IP) from H1299 cells transiently expressing the HA-Ubi. Proteins were detected by WB with the anti-V5 and anti-HA antibodies. Distinct HA-Ubi conjugates (1×Ubi to 4×Ubi) are indicated by their migration, based on the molecular weights of V5-pIX and HA-Ubi. **(E)** Antibody epitope tagging does not change pIX ubiquitination. V5 epitope-tagged ubiquitin (V5-Ubi) is specifically conjugated to the pIX(wt)-HA protein. Wild-type and mutant pIX-HA were immunopurified from H1299 cells transiently expressing V5-Ubi. Proteins were detected by WB with the anti-V5

and anti-HA antibodies. An asterisk indicates a potentially modified version of pIX, detected only in the case of pIX-HA.

Fig S4. pIX does not induce gene expression from its own promoter. A549 cells were transiently transfected with a plasmid containing the pIX promoter fused to the NanoLuc (NLuc) reporter gene together with plasmids expressing pIX(Wt)-HA or pIX(2Y>A)-HA. Relative NLuc expression was analyzed 24 h post-transfection from two independent experiments performed in technical triplicates. E1A expression was used as a positive control for pIX promoter activation.

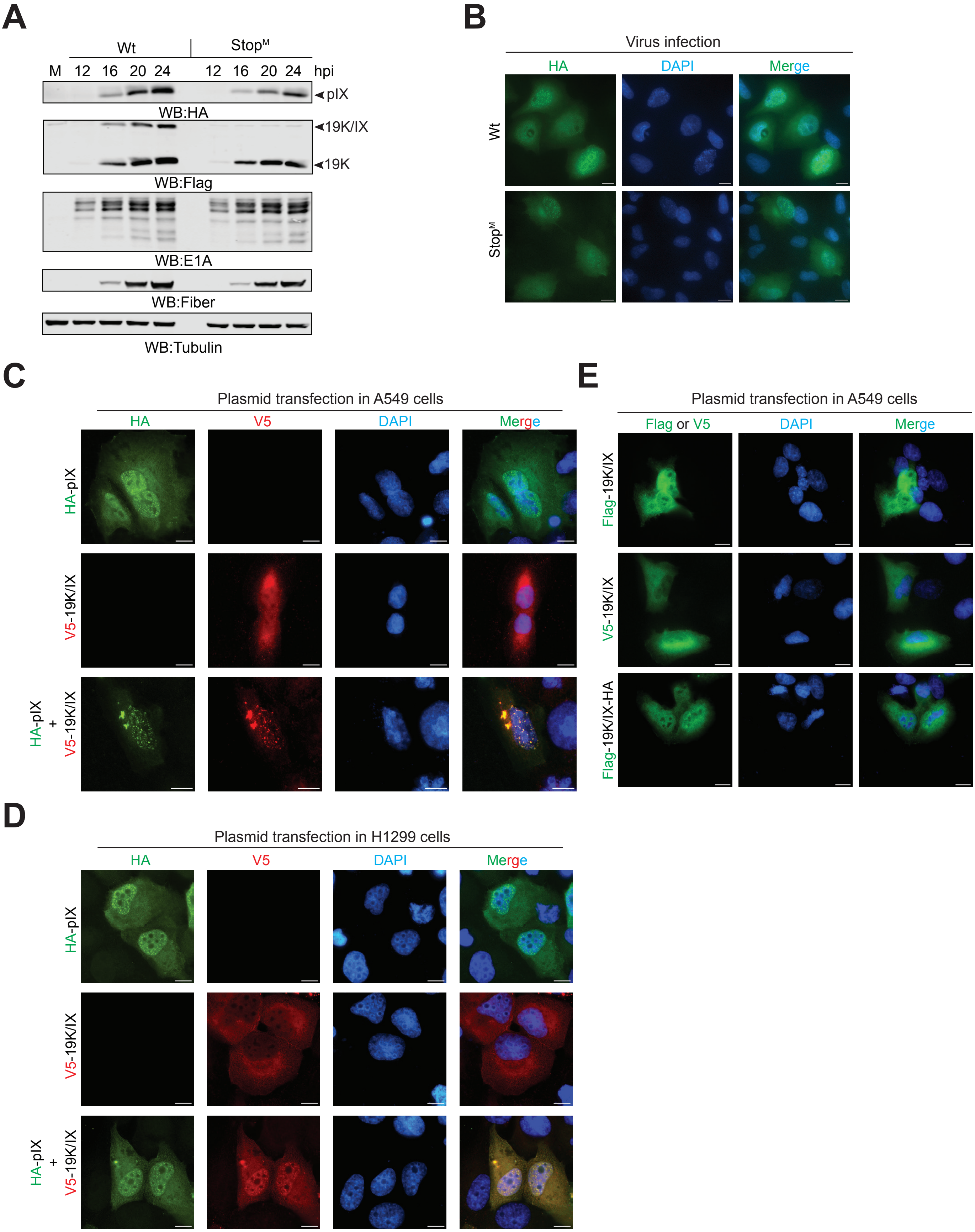
A
Wt
Stop^M
M 12 16 20 24 12 16 20 24 hpi
pIX
WB:HA
19K/IX
19K
WB:Flag
WB:E1A
WB:Fiber
WB:Tubulin
B
Virus infection
HA
DAPI
Merge
Wt
Stop^M
C
Plasmid transfection in A549 cells
HA
V5
DAPI
Merge
HA-pIX
V5-19K/IX
HA-pIX + V5-19K/IX
D
Plasmid transfection in H1299 cells
HA
V5
DAPI
Merge
HA-pIX
V5-19K/IX
HA-pIX + V5-19K/IX
E
Plasmid transfection in A549 cells
Flag or V5
DAPI
Merge
Flag-19K/IX
V5-19K/IX
Flag-19K/IX-HA

Fig. S1

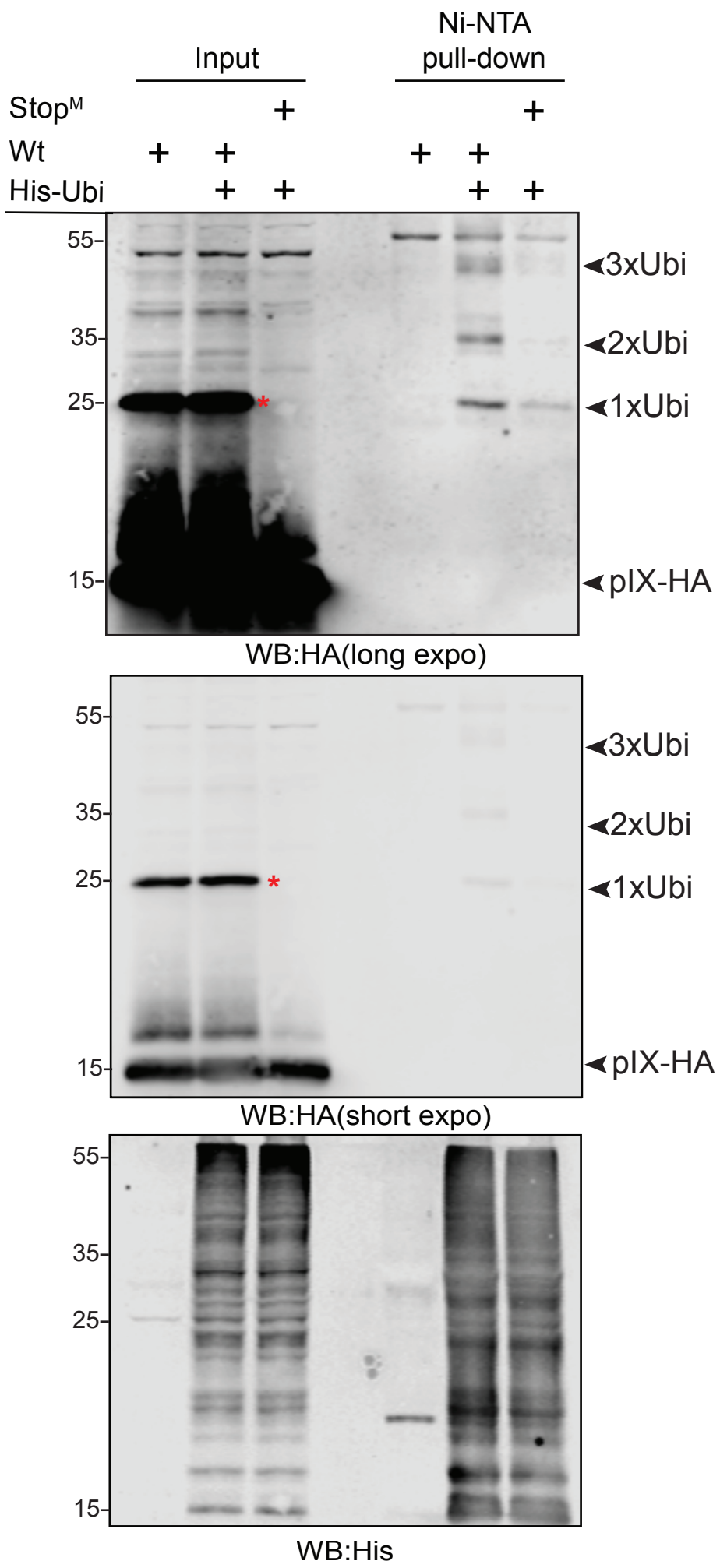
Input
Ni-NTA pull-down
Stop^M
Wt
His-Ubi
55-
35-
25-
15-
3xUbi
2xUbi
1xUbi
pIX-HA
WB:HA(long expo)
WB:HA(short expo)
WB:His

Fig. S2

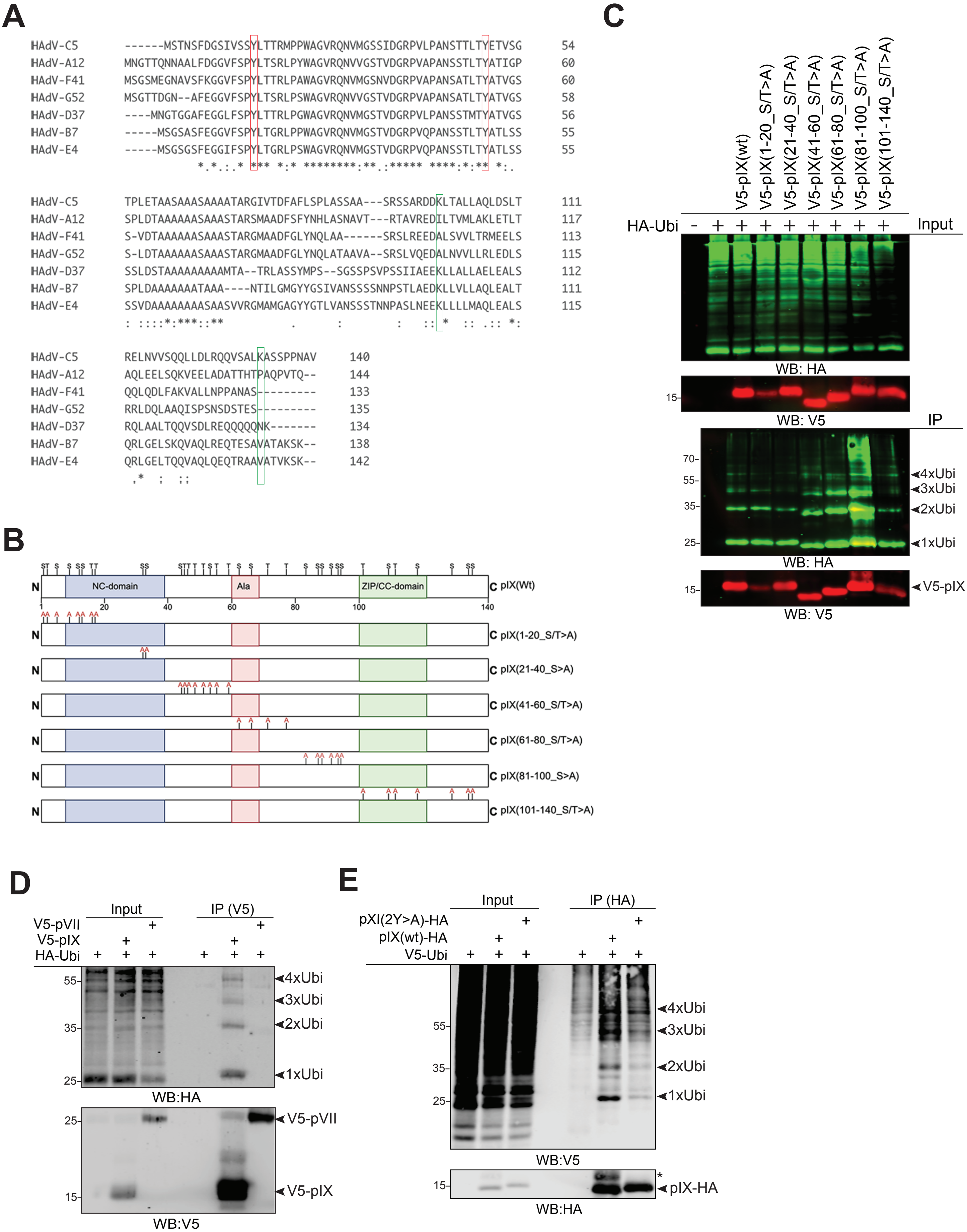
A
HAdV-C5 ------MSTNSFDGSIVSSYLTTRMPPWAGVRQNVMGSSIDGRPVLPANSTTLTYETVSG 54
HAdV-A12 MNGTTQNNAALFDGGVFSPYLTSRLPYWAGVRQNVVGSTVDGRPVAPANSSTLTYATIGP 60
HAdV-F41 MSGSMEGNAVSFKGGVFSPYLTTRLPAWAGVRQNVMGSNVDGRPVAPANSATLTYATVGS 60
HAdV-G52 MSGTTDGN--AFEGGVFSPYLTSRLPSWAGVRQNVVGSTVDGRPVAPANSATLTYATVGS 58
HAdV-D37 ----MNGTGGAFEGGLFSPYLTTRLPGWAGVRQNVMGSTVDGRPVLPANSSTMTYATVGS 56
HAdV-B7 -----MSGSASFEGGVFSPYLTGRLPPWAGVRQNVMGSTVDGRPVQPANSSTLTYATLSS 55
HAdV-E4 -----MSGSGSFEGGIFSPYLTGRLPSWAGVRQNVMGSTVDGRPVQPANSSTLTYATLSS 55
HAdV-C5 TPLETAASAAASAAAATARGIVTDFAFLSPLASSAA---SRSSARDDKLTALLAQLDSLT 111
HAdV-A12 SPLDTAAAAAASAAASTARSMAADFSFYNHLASNAVT---RTAVREDILTVMLAKLETLT 117
HAdV-F41 S-VDTAAAAAAASAAASTARGMAADFGLYNQLAA------SRSLREEDALSVVLTRMEELS 113
HAdV-G52 S-LDTAAAAAAASAAASTARGMAADFGLYNQLATAAVA--SRSLVQEDALNVVLLRLEDLS 115
HAdV-D37 SSLDSTAAAAAAAAAMTA--TRLASSYMPS--SGSSPSVPSSIIAEEKLLALLAELEALS 112
HAdV-B7 SPLDAAAAAAAATAAA----NTILGMGYYGSIVANSSSSNNPSTLAEDKLLVLLAQLEALT 111
HAdV-E4 SSVDAAAAAAAASAASVVRGMAMGAGYYGTLVANSSSTNNPASLNEEKLLLLMAQLEALS 115
HAdV-C5 RELNVVSQQLLDLRQQVSALKASSPPNAV 140
HAdV-A12 AQLEELSQKVEELADATTHTPAQPVTQ-- 144
HAdV-F41 QQLQDLFAKVALLNPPANAS--------- 133
HAdV-G52 RRLDQLAAQISPSNSDSTES--------- 135
HAdV-D37 RQLAALTQQVSDLREQQQQQNK------- 134
HAdV-B7 QRLGELSKQVAQLREQTESAVATAKSK-- 138
HAdV-E4 QRLGELTQQVAQLQEQTRAAVATVKSK-- 142
B
NC-domain
Ala
ZIP/CC-domain
pIX(Wt)
pIX(1-20_S/T>A)
pIX(21-40_S>A)
pIX(41-60_S/T>A)
pIX(61-80_S/T>A)
pIX(81-100_S>A)
pIX(101-140_S/T>A)
C
V5-pIX(wt)
V5-pIX(1-20_S/T>A)
V5-pIX(21-40_S/T>A)
V5-pIX(41-60_S/T>A)
V5-pIX(61-80_S/T>A)
V5-pIX(81-100_S/T>A)
V5-pIX(101-140_S/T>A)
HA-Ubi
Input
WB: HA
WB: V5
IP
4xUbi
3xUbi
2xUbi
1xUbi
V5-pIX
D
Input
IP (V5)
V5-pVII
V5-pIX
HA-Ubi
WB:HA
WB:V5
E
Input
IP (HA)
pXI(2Y>A)-HA
pIX(wt)-HA
V5-Ubi
pIX-HA

Fig. S3

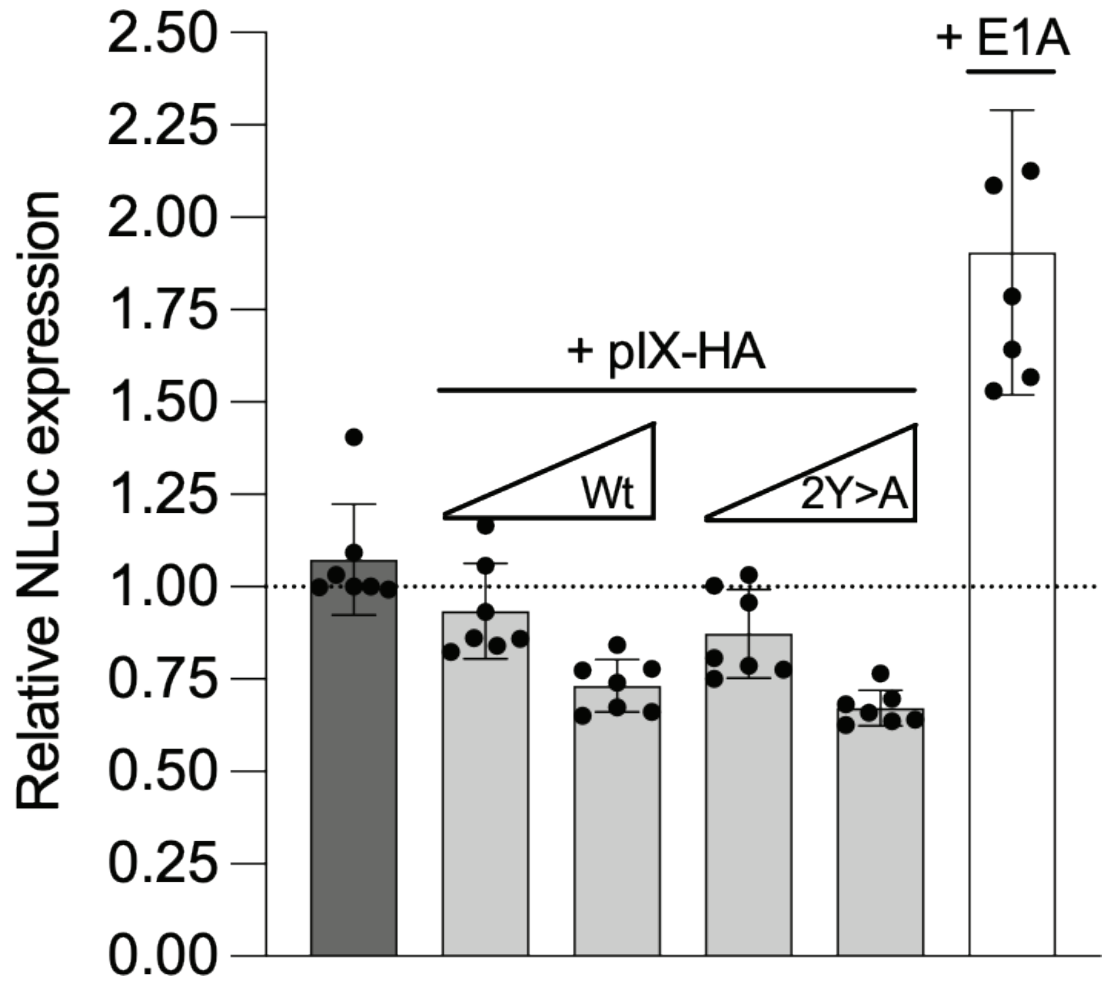
Relative NLuc expression
2.50
2.25
2.00
1.75
1.50
1.25
1.00
0.75
0.50
0.25
0.00
+ E1A
+ pIX-HA
Wt
2Y>A

Fig. S4
