# Supplementary material for: A novel adenovirus 19K/IX protein enhances infection by attenuating proteasomal degradation of tyrosine-ubiquitinated capsid protein IX
Source: mBio. 2026 Oct 14;17(11):e01683-26. doi: 10.1128/mbio.01683-26 (PMC13753832; doi:10.1128/mbio.01683-26)
Supplement: Supplementary file 2 [file mbio.01683-26-s0002.pdf]

Supplementary material and methods

Plasmid constructions

Synthetic HAdV-C5 pIX and 19K/IX cDNA fragments and their respective mutants (Twist Biosciences) were cloned into a pcDNA3 vector in-frame with the N-terminal Flag (Flag-pIX, Flag-19K/IX), HA (HA-pIX), V5 (V5-pIX, V5-19K/IX), or C-terminal HA (pIX-HA) antibody epitope tags (1, 2). Note that we used 19K/IX cDNA, not the E1B genomic region, to generate the different epitope-tagged 19K/IX expression constructs. The plasmids expressing PSMC3-Flag and PSMD9-Flag were constructed by cloning synthetic genes (NM_002804.4 and NM_002813.7, Twist Biosciences) into the pcDNA(C-Flag) vector background. Plasmid pRK5-HA-Ubiquitin was purchased from Addgene (#17608); the His-Ubiquitin-expressing plasmid has been described before (3). HA-Ubiquitin mutants K11R and K48R were generated by cloning the respective mutation-containing DNA fragment into the pRK5-HA-Ubiquitin vector background. The NanoLuc (pNL-pIX_promoter) reporter plasmid was generated by cloning the pIX promoter (4), corresponding to nucleotides 3510-3613 of HAdV-C5 (AC_000008.1), into the pNL1.1.PGK[*Nluc*/PGK] vector (Promega) using the KpnI and NcoI restriction enzymes. The HAdV-C5 E1A transcription unit (nucleotides 1-1740; AC_000008.1) was cloned into the pUC19 plasmid using the SalI and EcoRI restriction enzymes to express E1A proteins (pUC-E1A).

Isolation of the Adenobuilder viruses

Five days after transfection with assembled Adenobuilder genomes, cells underwent three freeze/thaw cycles to release viral particles. The resulting lysate was then used to infect HEK293AD or H1299 cells to amplify the virus. After 3 to 5 days of infection,

the cells were collected, resuspended in 400 μl of cell growth medium, and lysed through three freeze/thaw cycles on dry ice and at +37°C. Cell lysates were centrifuged at 5000 × g for 5 min at +4°C, and the virus-containing supernatants were aliquoted and stored at -80°C.

Virus titration

A549 cells were seeded into a 24-well plate and infected with virus dilutions ranging from 10^{-2} to 10^{-6}. At 40 hpi, cells were fixed in 4% paraformaldehyde (PFA) in Phosphate-Buffered Saline (PBS), then permeabilized with 0.125% Triton X-100/PBS (1). The cells were blocked in 2% BSA/PBS for 1 h at room temperature, then incubated overnight at +4°C with an anti-hexon antibody (TC31-9C12.C9-s, Developmental Studies Hybridoma Bank (DSHB), Table S3). Infected cells were detected with a fluorescently labeled secondary antibody (Alexa Fluor 488, A11001; Thermo Fisher Scientific). Fluorescent forming units (FFUs) were manually counted from appropriately diluted samples using a Nikon Eclipse TS100 microscope equipped for fluorescence detection at 10× magnification. Final virus titers (FFU/ml) were calculated using the dilution factor and the number of positively stained cells per well in a 24-well plate.

cDNA synthesis and qRT-PCR

Complementary DNA (cDNA) was generated using the Maxima reverse transcriptase (Thermo Fisher Scientific) primed with random hexamer primers (Thermo Fisher Scientific, SO142). Quantitative RT-PCR (qRT-PCR) reactions were prepared with FIREPol EvaGreen qPCR Supermix (Solis Biodyne, 08-36-00020) and amplified in a QuantStudio 6 Flex real-time PCR system (Thermo Fisher Scientific). Data from a

triplicate experiment were normalized to housekeeping genes (HRPT1), and relative mRNA expression (mean ± SD) was calculated using the ΔΔCt method. Primer sequences are available in Table S4.

Mass spectrometry

Analysis of MS raw files was performed using the FragPipe quantitative proteomics software suite (version 24.0), employing MSFragger (version 4.4.1) (5) for database searching and IonQuant (version 1.11.11) (6) for label-free quantification (LFQ). The LFQ-MBR (match-between-runs) workflow was used. Spectra were searched against the UniProtKB/Swiss-Prot *Homo sapiens* proteome (UP000005640, version 2024-11-21) and UniProtKB/Swiss-Prot human adenovirus C serotype 5 (UP000004992, version 2026-03-27) databases together with an in-house contaminant database. The precursor mass tolerance was set to 20 ppm, and the fragment mass tolerance to 0.6 Da. Up to two missed tryptic cleavages were allowed. Methionine oxidation and protein N-terminal acetylation were specified as variable modifications, and cysteine carbamidomethylation as a fixed modification. Peptide-spectrum matches (PSM) generated by MSFragger were rescored with MSBooster (version 1.4.14) (7) using AlphaPeptDeep-based prediction models for fragment ion spectra (AlphaPept MS2 Generic) and retention time (AlphaPept RT Generic) (8), followed by statistical validation with Percolator (version 3.7.1) (9). Protein inference was performed with ProteinProphet, and false discovery rate (FDR) filtering and report generation were carried out within the Philosopher-based FragPipe workflow (version 5.1.3-RC9) (10), with identifications filtered to 1% at the PSM, peptide, and protein levels. Downstream analysis was performed in FragPipe-Analyst (version 1.23) (11) using $\log_2$-transformed IonQuant protein intensities after removal of potential contaminant

proteins. Differential enrichment (DE) analysis was carried out using the limma framework, which fits feature-wise linear models and applies empirical Bayes moderation of the standard errors to obtain moderated *t*-statistics and associated *P*-values. Variance-stabilizing normalization (vsn) was applied. Missing values were imputed using Perseus-type imputation, in which missing values are replaced by random values drawn from a normal distribution with a downshift of 1.8 standard deviations and with a width of 0.3 for each sample. *P*-values were adjusted for multiple testing using the Benjamini-Hochberg method, and proteins with a $\log_2$ fold change of ≥2, and an adjusted *P*-value ≤0.05 were considered significantly enriched.

NanoLuc reporter assay

A549 cells grown in a 96-well plate were co-transfected with the pNL-pIX_promoter plasmid and increasing concentrations (20 ng and 80 ng) of the pIX(wt)-HA or pIX(Y2>A)-HA expressing plasmids. The total plasmid DNA concentration was kept the same (100 ng) in all the reactions with the empty pcDNA plasmid. E1A-expressing plasmid (pUC-E1A, 20 ng per well) was used as a positive control for pIX promoter activity. At 24 h post-transfection, the cells were lysed using the NanoGlo Luciferase Assay System (Promega), and the NanoLuc signal was detected with the Spark 10M plate reader (Tecan).

References

1. Schubert E, Mun K, Larsson M, Panagiotou S, Idevall-Hagren O, Svensson C, Punga T. 2024. Complex regulation of mitochondrial signaling by human adenovirus minor capsid protein VI. J Virol 98:e0035624.
2. Kases K, Schubert E, Hajikhezri Z, Larsson M, Devi P, Darweesh M, Andersson L, Akusjarvi G, Punga T, Younis S. 2023. The RNA-binding protein ZC3H11A interacts with the nuclear poly(A)-binding protein PABPN1 and alters polyadenylation of viral transcripts. J Biol Chem 299:104959.
3. Inturi R, Mun K, Singethan K, Schreiner S, Punga T. 2018. Human Adenovirus Infection Causes Cellular E3 Ubiquitin Ligase MKRN1 Degradation Involving the Viral Core Protein pVII. J Virol 92.
4. Babiss LE, Vales LD. 1991. Promoter of the Adenovirus Polypeptide-Ix Gene - Similarity to E1b and Inactivation by Substitution of the Simian Virus-40 Tata Element. Journal of Virology 65:598-605.
5. Kong AT, Leprevost FV, Avtonomov DM, Mellacheruvu D, Nesvizhskii AI. 2017. MSFragger: ultrafast and comprehensive peptide identification in mass spectrometry-based proteomics. Nature Methods 14:513-+.
6. Yu F, Haynes SE, Nesvizhskii AI. 2021. IonQuant Enables Accurate and Sensitive Label-Free Quantification With FDR-Controlled Match-Between-Runs. Mol Cell Proteomics 20:100077.
7. Yang KL, Yu FC, Teo GC, Li K, Demichev V, Ralser M, Nesvizhskii AI. 2023. MSBooster: improving peptide identification rates using deep learning-based features. Nature Communications 14.

8. Zeng WF, Zhou XX, Willems S, Ammar C, Wahle M, Bludau I, Voytik E, Strauss MT, Mann M. 2022. AlphaPeptDeep: a modular deep learning framework to predict peptide properties for proteomics. Nature Communications 13.
9. The M, MacCoss MJ, Noble WS, Käll L. 2016. Fast and Accurate Protein False Discovery Rates on Large-Scale Proteomics Data Sets with Percolator 3.0. Journal of the American Society for Mass Spectrometry 27:1719-1727.
10. Leprevost FD, Haynes SE, Avtonomov DM, Chang HY, Shanmugam AK, Mellacheruvu D, Kong AT, Nesvizhskii AI. 2020. Philosopher: a versatile toolkit for shotgun proteomics data analysis. Nature Methods 17:869-870.
11. Hsiao Y, Zhang H, Li GX, Deng Y, Yu F, Valipour Kahrood H, Steele JR, Schittenhelm RB, Nesvizhskii AI. 2024. Analysis and Visualization of Quantitative Proteomics Data Using FragPipe-Analyst. J Proteome Res 23:4303-4315.
