# Supplementary material for: A novel adenovirus 19K/IX protein enhances infection by attenuating proteasomal degradation of tyrosine-ubiquitinated capsid protein IX
Source: mBio. 2026 Oct 14;17(11):e01683-26. doi: 10.1128/mbio.01683-26 (PMC13753832; doi:10.1128/mbio.01683-26)
Supplement: Supplementary file 3 [file mbio.01683-26-s0003.pdf]

Table S1. Cloned plasmids used in the study

Name	Identifier	Purpose	Insert	Epitope tag
pcDNA-V5-19K/IX(Wt)	pTP907	WB/IF/IP	19K/IX(Wt)	N-terminal V5
pcDNA-Flag-pIX(Wt)	pTP886	WB/IF/IP	pIX(Wt)	N-terminal Flag
pcDNA-HA-pIX(Wt)	pTP902	WB/IF	pIX(Wt)	N-terminal HA
pcDNA-V5-pIX(Wt)	pTP970	WB/IF/IP	pIX(Wt)	N-terminal V5
pcDNA-HA-pIX(2K>R)	pTP955	WB	pIX(K99R/K132R)	N-terminal HA
pcDNA-V5-pIX(1-20_S/T>A)	pTP973	WB/IP	pIX(1-20_S/T>A)	N-terminal V5
pcDNA-V5-pIX(21-40_S/T>A)	pTP974	WB/IP	pIX(21-40_S/T>A)	N-terminal V5
pcDNA-V5-pIX(41-60_S/T>A)	pTP975	WB/IP	pIX(41-60_S/T>A)	N-terminal V5
pcDNA-V5-pIX(61-80_S/T>A)	pTP976	WB/IP	pIX(61-80_S/T>A)	N-terminal V5
pcDNA-V5-pIX(81-100_S/T>A)	pTP977	WB/IP	pIX(81-100_S/T>A)	N-terminal V5
pcDNA-V5-pIX(101-140_S/T>A)	pTP978	WB/IP	pIX(101-140_S/T>A)	N-terminal V5
pcDNA-V5-pIX(2Y>A)	pTP980	WB/IP	pIX(Y14A/Y49A)	N-terminal V5
pcDNA-V5-pIX(Y49A)	pTP1003	WB/IP	pIX(Y49A)	N-terminal V5
pcDNA-V5-19K/IX(2Y>A)	pTP1002	WB/IP	19K/IX(Y77A/Y112A)*	N-terminal V5
pcDNA-pIX(Wt)-HA	pTP1018	WB/IF/IP/NLuc	pIX(Wt)	C-terminal HA
pcDNA-pIX(2Y>A)-HA	pTP1022	WB/IF/IP/NLuc	pIX(Y14A/Y49A)	C-terminal HA
pcDNA-pIX(Y14A)-HA	pTP1029	IF	pIX(Y14A)	C-terminal HA
pcDNA-pIX(Y49A)-HA	pTP1030	IF	pIX(Y49A)	C-terminal HA
pcDNA-PSMC3-Flag	pTP1015	WB/IF	PSMC3	C-terminal Flag
pcDNA-PSMD9-Flag	pTP1016	WB/IF	PSMD9	C-terminal Flag
pRK5-HA-Ubi(Wt)	pTP994	WB/IP	Ubiquitin(Wt)	N-terminal HA
pRK5-HA-Ubi(K11R)	pTP1043	WB/IP	Ubiquitin(K11R)	N-terminal HA
pRK5-HA-Ubi(K48R)	pTP1044	WB/IP	Ubiquitin(K48R)	N-terminal HA
pRK5-HA-Ubi(K11R/K48R)	pTP1045	WB/IP	Ubiquitin(K11R/K48R)	N-terminal HA
pcDNA-V5-pVII(K26R/K27R)	pTP979	WB/IP	pVII(K26R/K27R)	N-terminal V5
pUC-E1A	pTP699	NLuc	E1A(nt. 1-1740)	N/A
pNL-pIX_promoter	pTP1028	NLuc	pIX (nt. 3510-3613)	N/A

*The point mutations Y14A/Y49A in pIX correspond to amino acids Y77A/Y112A in the 19K/IX protein, due to 63-amino-acid N-terminal extension from E1B19K CDS.

WB; Western blot, IF; immunofluorescence, IP; immunoprecipitation, NLuc; NanoLuc reporter gene assay

Table S2. Adenobuilder-based viruses used in the study

**Virus name**	**Epitope tagged and modified virus genes**
Wt_AB	unmodified AdenoBuilder-based (1) wild-type HAdV-C5 expressing no epitope-tagged proteins
Wt	**Flag**-E1B19K<br>pIX-**HA**<br>**Flag**-19K/IX-**HA**
Stop^{M}	**Flag**-E1B19K<br>pIX-**HA**
5'ss^{M}	**Flag**-E1B19K<br>pIX-**HA**
Wt_Gal4	**Flag**-E1B19K<br>pIX-**HA**<br>**Flag**-19K/IX-**HA**<br>**V5**-Gal4
Stop^{M}_Gal4	**Flag**-E1B19K<br>pIX-**HA**<br>**V5**-Gal4
Stop^{M}_V5-19K/IX	**Flag**-E1B19K<br>pIX-**HA**<br>**V5**-19K/IX
Wt(2K>R)	**Flag**-E1B19K<br>pIX(K99R/K132R)-**HA**<br>**Flag**-19K/IX(K162R/K195R)-**HA***
Stop^{M}(2K>R)	**Flag**-E1B19K<br>pIX(K99R/K132R)-**HA**
Wt(2Y>A)	**Flag**-E1B19K<br>pIX(Y14A/Y49A)-**HA**<br>**Flag**-19K/IX(Y77A/Y112A)-**HA***
Stop^{M} (2Y>A)	**Flag**-E1B19K<br>pIX(Y14A/Y49A)-**HA**

***** The point mutations in pIX are also present in the 19K/IX protein, which contains a 63-amino-acid N-terminal extension.

1. Jang Y, Bunz F. 2022. AdenoBuilder: A platform for the modular assembly of recombinant adenoviruses. Star Protocols 3.

Table S3. Antibodies used for western blot, immunofluorescence, and virus titer assays

Target	Dilution	Purpose	Host	Source	Identifier/reference
Anti-pV	1:5000	WB	Rabbit	D.Matthews	(1)
Anti-E1B55K (2A6)	1:40	WB	Mouse	T.Dobner	(2)
Anti-AVP	1:100	WB	Rabbit	M.Balakirev	(3)
Anti-DBP	1:500	IF	Mouse	A.Levine	A72K A1-6, (4)
Anti-GAPDH (FL-335)	1:1000	WB	Rabbit	Santa Cruz	sc-25778
Anti-Tubulin (6A204)	1:1000	WB	Mouse	Santa Cruz	sc-69969
Anti-Gal4 DBD	1:1000	WB	Mouse	Santa Cruz	sc-510
Anti-E1A(M58)	1:1000	WB/IF	Mouse	Santa Cruz	sc-58658
Anti-V5	1:1000	WB/IF	Rabbit	Proteintech	14440-1-AP
Anti-CoxIV	1:1500	WB	Rabbit	Proteintech	11242-1-AP
Anti-HA	1:1000	WB/IF	Rabbit	Proteintech	51064-2-AP
Anti-Flag	1:1000	WB/IF	Rabbit	Proteintech	12552-1-AP
Anti-PSMC3	1:1000	WB	Rabbit	Proteintech	24142-1-AP
Anti-PSMD9	1:1000	WB	Rabbit	Proteintech	26922-1-AP
Anti-Flag	1:1000	WB/IF	Mouse	Proteintech	66008-4-IG
Anti-V5	1:1000	WB	Mouse	Proteintech/Chromotek	SV5-P-K
Anti-Adenovirus type 5	1:2000	WB	Rabbit	Abcam	Ab6982
Anti-Histone H3	1:30.000	WB	Rabbit	Abcam	ab1791
Anti-HSPA1A	1:1000	WB	Mouse	Thermo	MA3-006
Anti-His	1:1000	WB	Mouse	Takara	631212
Anti-Hexon	1:100	Titration	Mouse	DSHB	TC21-9C12-C9
Anti-Fiber	1:2000	WB	Mouse	Invitrogen	MA5-11222
Anti-HA.11	1:1000	WB/IF	Mouse	BioLegend	901513
Anti-HA	1:1000	WB	Rabbit	Sigma	H6908

WB; Western blot, IF; immunofluorescence, Titration; virus titration

References

1. Matthews DA, Russell WC. 1998. Adenovirus core protein V is delivered by the invading virus to the nucleus of the infected cell and later in infection is associated with nucleoli. Journal of General Virology 79:1671-1675.
2. Sarnow P, Sullivan CA, Levine AJ. 1982. A Monoclonal-Antibody Detecting the Adenovirus Type-5 E1b-58kd Tumor-Antigen - Characterization of the E1b-58kd Tumor-Antigen in Adenovirus-Infected and Adenovirus-Transformed Cells. Virology 120:510-517.
3. Balakirev MY, Jaquinod M, Haas AL, Chroboczek J. 2002. Deubiquitinating function of adenovirus proteinase. Journal of Virology 76:6323-6331.
4. Reich NC, Sarnow P, Duprey E, Levine AJ. 1983. Monoclonal-Antibodies Which Recognize Native and Denatured Forms of the Adenovirus DNA-Binding Protein. Virology 128:480-484.

Table S4. PCR/qPCR primers to detect host cell and viral mRNAs

Target	Forward	Reverse	Purpose
HAdV-C5 Flag-19K/IX-HA	5'-GCG*GAATTC*GCCACC**ATGGACTACAAGG**-3'	5'-TGC*CTCGAG*TCATTA**AGCGTAATC**-3'	Viral mRNA detection by endpoint PCR
GAPDH	5'-GGTTTACATGTTCCAATATGATTCCA-3'	5'-ATGGGATTTCCATTGATGACAAG-3'	Housekeeping gene mRNA detection by endpoint PCR
HRPT1	5'- TGGACAGGACTGAACGTCTTG -3'	5'- CCAGCAGGTCAGCAAAGAATTTA-3'	Housekeeping gene mRNA detection by qRT-PCR
HAdV-C5 E1A	5'- GTGCCCCATTAAACCAGTTG-3'	5'- GGCGTTTACAGCTCAAGTCC-3'	Viral mRNA detection by qRT-PCR
HAdV-C5 pIX	5'-CCGCGATGACAAGTTGACGG-3'	5'-CATTGGGAGGGGAGGAAGCC-3'	Viral mRNA detection by qRT-PCR

Primer 5’-GCG*GAATTC*GCCACC**ATGGACTACAAGG**-3' contains an EcoRI restriction site (Italics) and a sequence complementary to the Flag antibody epitope-coding sequence (bold)

Primer 5'-TGC*CTCGAG*TCATTA**AGCGTAATC**-3' contains an XhoI restriction site (Italics) and a sequence complementary to the HA antibody epitope-coding sequence (bold)

The primer pair (5′-CCGCGATGACAAGTTGACGG-3′ and 5′-CATTGGGAGGGGAGGAAGCC-3′) amplifies pIX CDS corresponding to nucleotides 3893–4023 of the HAdV-C5 genome.

The primer pair (5’-GCG*GAATTC*GCCACC**ATGGACTACAAGG**-3' and 5'-TGC*CTCGAG*TCATTA**AGCGTAATC**-3') amplifies only the Flag-19K/IX-HA transcript and not the Flag-E1B19K or pIX-HA transcripts.
